# Obliteration of the Œsophagus

**Published:** 1847-02

**Authors:** 


*Obliteration of the Œsophagus.*—Prof. Levy, of Copenhagen, relates two cases of this malformation, in the *Neue Zeitschrift fur Geburtskunde.* The first case was that of an infant, apparently healthy when born, but which was unable to suck, and rejected everything that was administered to it. The day after birth a catheter was introduced into the œsophagus, but was arrested at a depth of four inches. Milk injected through the catheter was immediately returned by the nose and mouth, producing symptoms of suffocation. Closure of the œsophagus was accordingly diagnosticated, and some broth was injected into the rectum. At the period of its birth, the infant weighed six pounds and a half; forty-six hours afterwards it only weighed five pounds and a half; fifty-eight hours after, it weighed five pounds and a quarter; seventy hours after birth, it weighed five pounds and an eighth; eighty-two hours after, five pounds. The infant died eighty-three hours after birth. The child lost, therefore, during four days, one pound and a half in weight, half-a-pound being the loss during the last two days. The urine and feces presented a natural appearance; they were passed at long intervals, and scarcely exceeded the weight of the broth injected. On post-mortem examination, the upper portion of the œsophagus was found to terminate in a cul-de-sac, in which the catheter introduced by the mouth, was arrested. This sac was about an inch long, and twice the size of an ordinary œsophagus. The structure of the muscular fibres presented nothing abnormal, except that they converged towards the truncated extremity of the tube. The inferior portion of the œsophagus was connected with the trachea and larynx; so that a catheter introduced through an incision in the cardiac extremity of the stomach, passed through the trachea and larynx into the mouth. The communication between the trachea and the œsophagus took place about a line below the bifurcation of the trachea, and was formed by an oval opening, three lines long and one line wide. The posterior wall of the air-duct and the respiratory mucous membrane were continuous with those of the digestive canal. In the second case, the fœtus was born before the full period of gestation, and died immediately after birth. In this case, Profesor Levy found, besides several malformations in the region of the pelvis, that the upper part of the œsophagus terminated in a cul-de-sac, and that the lower communicated with the right bronchus. The communication of the tube with the trachea differed from that in the former case, inasmuch as the œsophagus contained some cartilaginous rings in a part of its extent. With the exception of the case of Martin, republished in the pathological anatomy of M. Andral, Professor Levy has never seen any other examples of this malfomation than those just related. These cases support the opinion of those who regard monstrosities as arrests of development.—*Med. Times.*

